# Localized expression and inhibition effect of miR-184 on blood digestion and oviposition in *Haemaphysalis longicornis* (Acari: Ixodidae)

**DOI:** 10.1186/s13071-019-3754-7

**Published:** 2019-10-25

**Authors:** Muhammad Irfan Malik, Mohsin Nawaz, Yanan Wang, Houshuang Zhang, Jie Cao, Yongzhi Zhou, Ibrahim A. Hassan, Md. Nazrul Islam, Muhammad Naveed Anwar, Jinlin Zhou

**Affiliations:** Key Laboratory of Animal Parasitology of Ministry of Agriculture, Shanghai Veterinary Research Institute, Chinese Academy of Agricultural Sciences, Shanghai, 200241 China

**Keywords:** *Haemaphysalis longicornis*, miR-184, Vitellogenin, Blood digestion, Oviposition

## Abstract

**Background:**

The hard tick *Haemaphysalis longicornis* (Ixodidae) is widely distributed in East Asia, China, Australia and New Zealand. It can transmit many infectious pathogens, including the causative agents of human rickettsiosis, bovine theileriosis, bovine babesiosis and canine babesiosis. Therefore, a greater understanding of *H. longicornis* biology might aid in the development of more effective control measures against the tick and tick-borne pathogens.

**Methods:**

We analyzed the expression of miR-184 in different developmental stages and various tissues of *H. longicornis* using real-time PCR (qRT-PCR). Antagomir (Ant-184) was used to knock-down miR-184, whilst Ms-Ant and non-injected ticks were used as the negative and blank controls, respectively. We used online software tools (RNAhybrid and TargetScan) to predict the putative target genes of miR-184.

**Results:**

The expression of miR-184 was highest in unfed nymphs and lowest in unfed larvae. The tissue distribution of miR-184 showed abundant expression in the midgut. To investigate the probable roles of miR-184, antagomir (Ant-184) was used to knock-down miR-184 (*t*_(4)_ = 12.32, *P* = 0.0002). After inhibiting miR-184, other biological factors were examined in each group. The engorged body weight was significantly reduced in the treated group (Ant-184) in contrast to control groups (*t*_(22)_ = 2.19, *P* = 0.0388). The mean duration of the egg-laying days was significantly increased (33.5 ± 1.91) and the number of eggs (*t*_(10)_ = 3.147, *P* = 0.0137), and egg mass (*t*_(10)_ = 3.4472, *P* = 0.0063) were significantly reduced in the treated group. During oviposition, eggs were monitored and in half of the ticks of the Ant-184 group the eggs were completely desiccated, lacked embryo development and did not hatch. We analyzed the expression of Vg proteins (Vg1, Vg2, Vg3) in semi-engorged ticks, engorged ticks, ticks at day 2 after engorgement and egg stage in Ant-184, non-injected and Ms-Ant groups, and found significant variation.

**Conclusions:**

This study provides information on the role of miR-184 in *H. longicornis* ticks. The data suggest that miR-184 targets Vg proteins and affects blood digestion and oviposition.

## Background

Tick species are widely distributed throughout the world, especially in tropical and subtropical regions [1]. They transmit various pathogens of medical and veterinary importance such as bacteria, rickettsiae, protozoans, helminths, spirochaetes and viruses [2]. The identification of new pathogens illustrates the threat of tick-borne diseases. In the last 30 years, tick-borne pathogens have become an increasing hazard to human health worldwide.

MicroRNAs (miRNAs) are short non-coding RNAs ranging from 19 to 24 nucleotides in length. They function as modulators of gene expression at the post-transcriptional level by binding, *via* base-pairing, to their target mRNA. They are important in the development, differentiation, proliferation and apoptosis of organisms and cancers in animals, viruses and plants [3–6]. Some miRNAs have tissue- or development-specific expression, with precise and distinct functions. miR-184, with only a one copy number, is evolutionarily preserved from *Drosophila* to humans at the nucleotide level. In *Drosophila*, miR-184 is expressed in the mesoderm and it has several functions in female germline development [7]. It is expressed in the epidermis, lens and the hatching gland in the zebrafish (*Danio rerio*) [8]. miR-184 has also been observed to be expressed in the brain and testes of mice under the control of MeCP2 [9].

Vitellogenin (Vg) is a phospho-lipoglyco protein and the precursor of vitellin [10]. It is usually present in oviparous animals. In ticks, Vg is regulated by 20-hydroxyecdysone. It is primarily synthesized in the fat body and transported, after a blood meal, to specific tissues [11, 12]. Tick Vg is present in eggs, larvae and fed adults. During embryonic development, Vg provides carbohydrates, amino acids and other nutrients. There are several Vg forms in different tissues of *Haemaphysalis longicornis*. In honeybees, Vg may affect foraging behavior and modulate the expression of miRNAs [13]. Vg is also associated with miRNA expression and, in zebrafish, six distinctively expressed miRNAs interact with specific Vg proteins [14].

Although various tick-specific miRNAs have been identified [15], *in vivo* functional studies have not been reported. miR-184 may be one of the top ten most abundantly expressed miRNA in tick saliva [16] and it is highly expressed in adult (fed and unfed) *H. longicornis* [17]. There is no information about the functional role of miR-184 in *H. longicornis*.

The hard tick *H. longicornis* is widely distributed in East Asia, China, Australia and New Zealand. It transmits many infectious pathogens, including the causative agents of human rickettsiosis, bovine theileriosis, bovine babesiosis and canine babesiosis [18, 19]. A greater understanding of *H. longicornis* biology might aid in the development of more effective control measures.

In the present study, we determined the expression profile and function of miR-184 in *H. longicornis.*

## Methods

### Computational prediction of miRNA targets

We used online software tools (RNAhybrid and TargetScan) to predict the putative target genes of miR-184. We selected four identified Vg proteins (Vg1, Vg2, Vg3 and VgB) with sequences available on GenBank. Only the first three were analyzed because the functions of VgB are currently unspecified in *H. longicornis*.

### Tick rearing and feeding

A colony of *H. longicornis* ticks was maintained by feeding on rabbits in our research laboratory at the Chinese Academy of Agricultural Sciences Shanghai, China [17]. Ears of New Zealand rabbits were used for blood-feeding and ticks were kept in cloth bags until they were semi-engorged or fully-engorged. The ticks were recollected after feeding, washed with ethanol (75%) and used for sample collection.

### Sample collection at different growth phases and tissue dissection

Samples were extracted at different growth phases (eggs, larvae, nymphs and adults) from both unfed and fed stages of ticks. Unfed and semi-engorged adult ticks were dissected and tissue samples (midguts, salivary glands and ovaries) were collected as previously described [20]. Briefly, the ticks were placed under a dissection light microscope and submerged in ice-cold phosphate-buffered saline (PBS; autoclaved; pH 7.4). While the ticks were held with a pair of soft-tissue forceps, the dorsal cuticle was removed and the various tissues (midgut, salivary glands and ovary) were separated using an 18-gauge needle. Then, tissues were rinsed with PBS, placed into micro-centrifuge tubes and stored at − 80 °C. The samples obtained from various growth phases of ticks were crushed in a mortar with a pestle using liquid nitrogen (− 196 °C). Then, TRIzol (Invitrogen, Carlsbad, CA, USA) was added to the samples and they were stored at − 80 °C.

### Total RNA isolation

The total RNA was isolated from different growth phases and tissues of ticks using TRIzol reagent according to manufacturer’s instructions (Invitrogen, Carlsbad, CA, USA). The concentration of the extracted RNA was measured using a Bio Photometer (GE, Fairfield, CT, USA), while the purity and integrity were determined by standard agarose gel electrophoresis.

The RNA samples were stored at − 80 °C.

### Real-time PCR

miR-184 cDNA, for quantitative (q) RT-PCR, was transcribed using Hiflex buffer (miScript II RT kit, Qiagen, Hilden, Germany) according to the manufacturer’s protocol. The miScript SYBR Green PCR Kit (Qiagen) including universal reverse primer and the forward primer (5′-TGG ACG GAG AAC TGA TAA GGG-3′) were used to amplify miR-184, and the tick elongation factor 1α (ELF1A) was used as the internal control. The real-time PCR was performed on an Applied Biosystems 7500 (Waltham, MA, USA), using the miScript SYBR Green PCR Kit.

The cDNA for Vg proteins was transcribed with a Prime Script RT reagent kit (Takara, Shinga, Japan) using manufacturer’s instructions. The relative expression of predicted target Vg proteins was analyzed by real-time PCR performed on an Applied Biosystems 7500 using SYBR Premix Ex Taq II (Tli RNaseH plus). Tick elongation factor 1α (ELF1A) was used as the internal control. The primers used for qRT-PCR are given in Table 1. Each reaction was executed in triplicate and relative expression of miRNA and Vg proteins was calculated using the 2^−∆Ct^ method.

**Table 1:** Primers used for qRT-PCR

Vg	Forward primer (5′-3′)	Reverse primer (5′-3′)
Vg1	CTCACGGCCGACATCGAATA	GTTGGTCACGTCGAAGTCCT
Vg2	CCGCCACCATGAGAATCGTA	CGTCAGTTGCTGAGGATGGT
Vg3	ATCAATCCACAAGGCAGCGA	ATCTTGGTCACTTCCAGCCG

### Feeding periods and duration of egg-laying

The feeding periods for each tick group were measured using the formula: Number of ticks engorged × Feeding days / Total number of ticks. The duration of egg-laying was calculated using the method described by Hadi & Adventini [21]. This was: Average duration of egg-laying = (egg-laying start − egg-laying stop)/Total number of egg-laying ticks.

### Antagomir synthesis and application

Antagomir is a chemically modified single-stranded miRNA, specifically tagged and designed from a mature miRNA sequence that was used for the silencing of miR-184. Antagomir 3′ terminal was modified by cholesterol and there were two thiols modified in the 5′ terminal, four thiols modified in the 3′ terminal, and the whole strand was modified by 2′-Ome. The missense antagomir (Ms-Ant) is chemically modified and optimized nucleic acid used as a negative control. These were purchased from GenePharma (Shanghai, China; http://www.genepharma.com). Non-injected ticks were used as a blank control. Antagomir and Ms-Ant were injected using an IM 300 microinjector (Narishige, Tokyo, Japan) at a dose of 0.5 μl (10 μM) through the joint between the IV coxa and the sternal plate of 50 unfed adult *H. longicornis*. Microinjected ticks were kept in an incubator for 24 h at 25 °C and 92% relative humidity, RH. Each group contained 38 ticks that were placed on a separate rabbit for blood feeding.

### Statistical analysis

All data were analyzed with GraphPad Prism 6 using Student’s t-test. The results are shown as the mean ± standard error of the mean, SEM (*P <* 0.05 was considered as significant).

## Results

### Expression profile of miR-184 in various growth phases and tissues

**Fig. 1:**
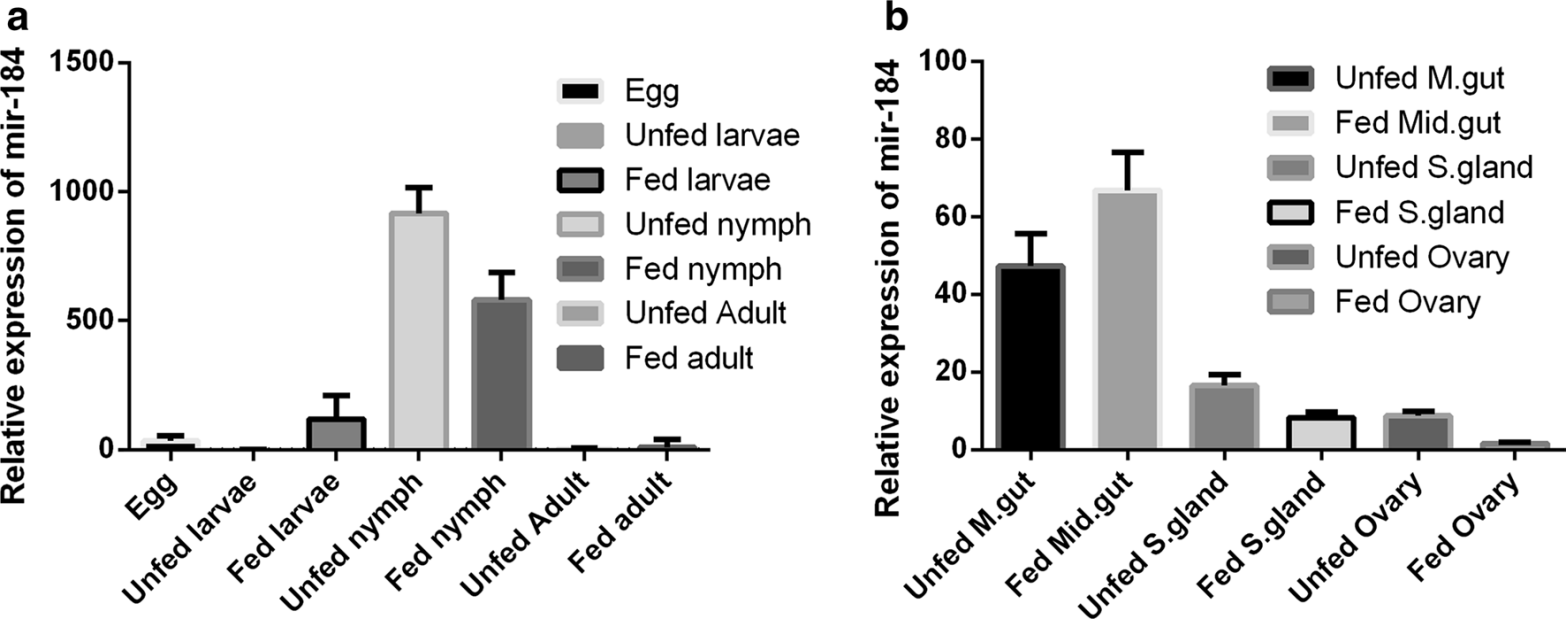
miR-184 expression in different developmental stages and tissues. **a** Relative expression of miR-184 was analyzed in eggs, unfed larvae, fed larvae, unfed nymphs, fed nymphs, unfed adults and fed adults. **b** Relative expression of miR-184 in midgut, salivary gland and ovary in unfed and fed female ticks. Data represent three biological replicates with three technical replicates and are shown as the mean ± SEM

We analyzed the potential biological roles of miR-184 in *H. longicornis.* We measured the expression level using real-time PCR in different growth phases (egg, larva, nymph and adult) in both unfed and fed stages and in various tissues (midgut, salivary gland and ovary). Expression in the different growth phases was significantly greater in unfed and fed nymphs, but lower in other stages (Fig. 1a). The relative expression of miR-184 in various tissues was highest in the midgut (increased in fed adults compared to unfed adults) and lowest in other tissues (Fig. 1b).

### Silencing of miRNA-184 affects blood digestion and oviposition

**Fig. 2:**
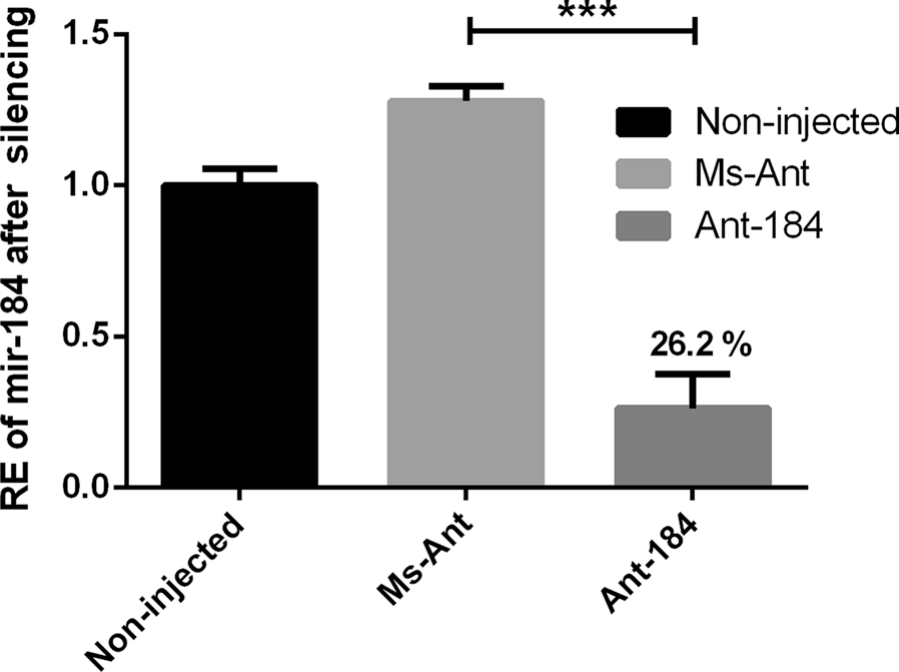
Relative miR-184 expression in the fed adult ticks. The miR-184 inhibited by Antagomir-184. Data represent three biological replicates with three technical replicates and are shown as the mean ± SEM

To study the role of miR-184 in adult female *H. longicornis*, the sequence-specific antagomir (Ant-184) was used to inhibit miR-184. Silencing efficiency on day 4 post-feeding was calculated using quantitative real-time PCR. The expression level of miR-184 was successfully reduced to 26.2% in the Ant-184 treated group as compared to the Ms-Ant and non-injected control groups (Fig. 2; *t*_(4)_ = 12.32, *P* = 0.0002).

**Fig. 3:**
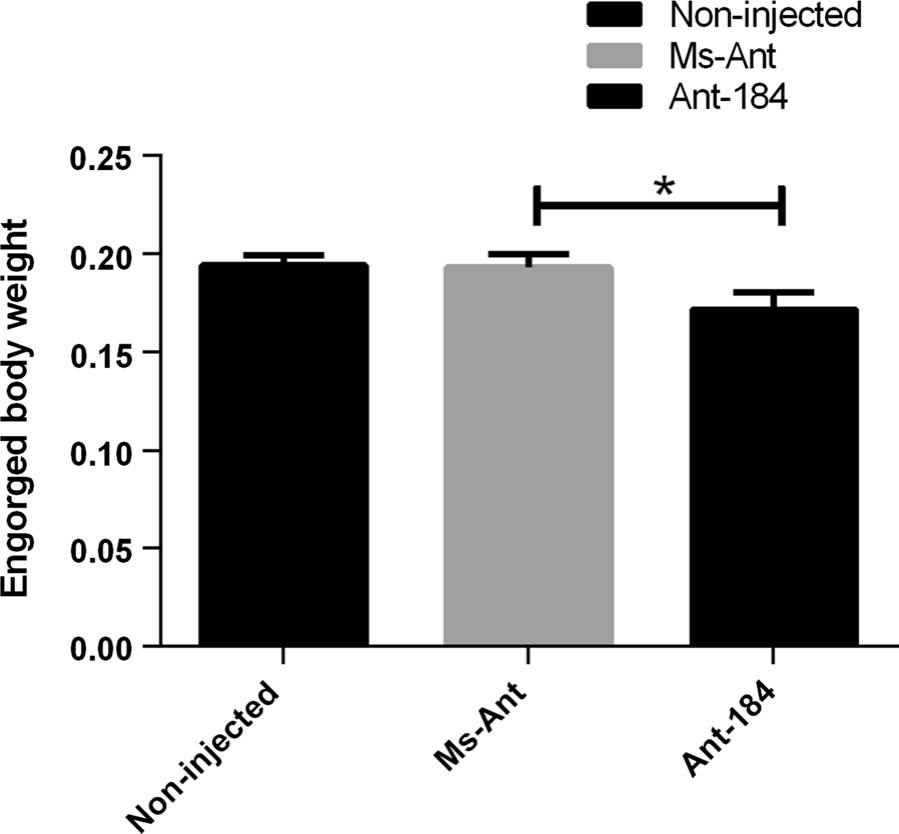
Average engorged weight after silencing in treated and control groups. Data represent three biological replicates with three technical replicates and are shown as the mean ± SEM

After inhibition of miR-184, several biological factors were studied in each group. The engorged body weight was significantly reduced in treated group Ant-184 compared to the control groups (Fig. 3; *t*_(22)_ = 2.19, *P* = 0.0388).

**Fig. 4:**
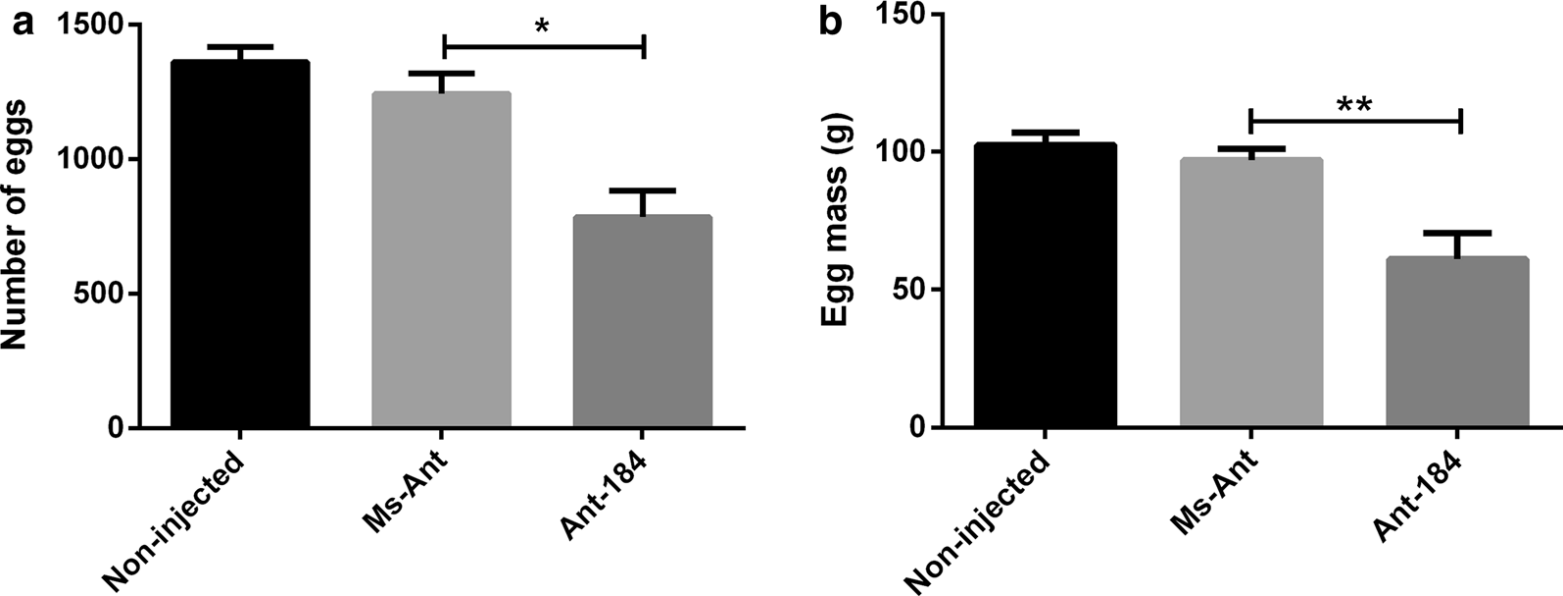
Silencing of miR-184 affects blood digestion. Average numbers of eggs (**a**) and egg mass (**b**) in non-injected ticks, Ms-Ant and Ant-184 ticks. The data are shown as the mean ± SEM

The majority of *H. longicornis* females began to lay eggs approximately 6 days after engorgement. The mean numbers of eggs were 1361, 1243 and 843, in non-injected, Ms-Ant and Ant-184 groups, respectively (Fig. 4a; *t*_(10)_ = 3.147, *P* = 0.0137).

The average egg mass was significantly reduced in Ant-184 (60.94 ± 9.569), Ms-Ant (96.69 ± 4.03) and non-injected control (102.45 ± 4.54) (Fig. 4b; *t*_(10)_ = 3.4472, *P* = 0.0063).

The average egg-laying duration was significantly longer (33.5 ± 1.91 d) in Ant-184, compared to 27.8 ± 1.24 d in Ms-Ant and 26.4 ± 1.02 d for the non-injected group (*t*_(10)_ = 2.543, *P* = 0.0345; Table 2).

**Table 2:** Feeding period and egg-laying duration in different groups

Group	Feeding period (days)	Egg-laying duration (days)
Non-injected	6.3	26.4 ± 1.02
Ms-Ant	6.0	27.8 ± 1.24
Ant-184	5.6	33.5 ± 1.91*

*P < 0.05

**Fig. 5:**
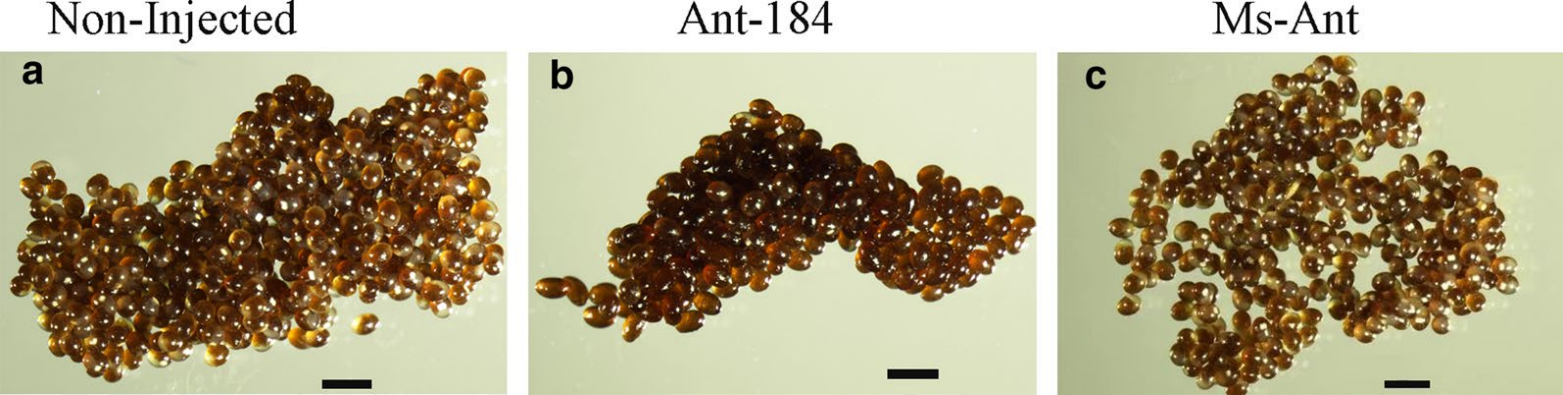
miR-184 inhibition affects egg development. Non-injected (**a**) and Ms-Ant control (**c**) showed normal embryo development, whereas in the Ant-184-treated group (**b**) eggs became desiccated and lacked development. *Scale-bars*: 1000 µm

Eggs were examined during oviposition in the control and treated groups (Fig. 5). The eggs in approximately 50% of ticks in the Ant-184 group became desiccated and lacked embryo development (Fig. 5b) and did not hatch (Table 3). The final weight and change rate of engorged weight showed no significant difference in treated and control groups (Table 4).

**Table 3:** Inhibition of miR-184 affects egg hatching

Group	Total no. of ticks	No. of ticks with egg hatching
Non-injected	12	12
Ms-Ant	12	12
Ant-184	12	6*

*P < 0.05

**Table 4:** The effect of Ant-184 on the rate of change of engorged and final weight

Group	Rate of change of engorged weight (%)^a^	Final weight (mg)^b^
Non-injected	73.3 ± 1.31	51.6 ± 0.30
Ms-Ant	74.2 ± 1.57	52.1 ± 0.39.
Ant-184	62.7 ± 0.56	66.6 ± 1.05

^a^The percentage of rate of change of engorged weight was calculated as: (engorged weight − final weight)/engorged weight

^b^Final weight was the weight on day 30 after engorgement (female ticks stop laying and die)

### Expression analysis of vitellogenin

To determine the potential targets involved in the silencing effect of miR-184, we used qRT-PCR to study the three known *H. longicornis* Vg proteins (Vg1, Vg2, Vg3) in semi-engorged ticks, engorged ticks, ticks at day 2 after engorgement and egg stage.

**Fig. 6:**
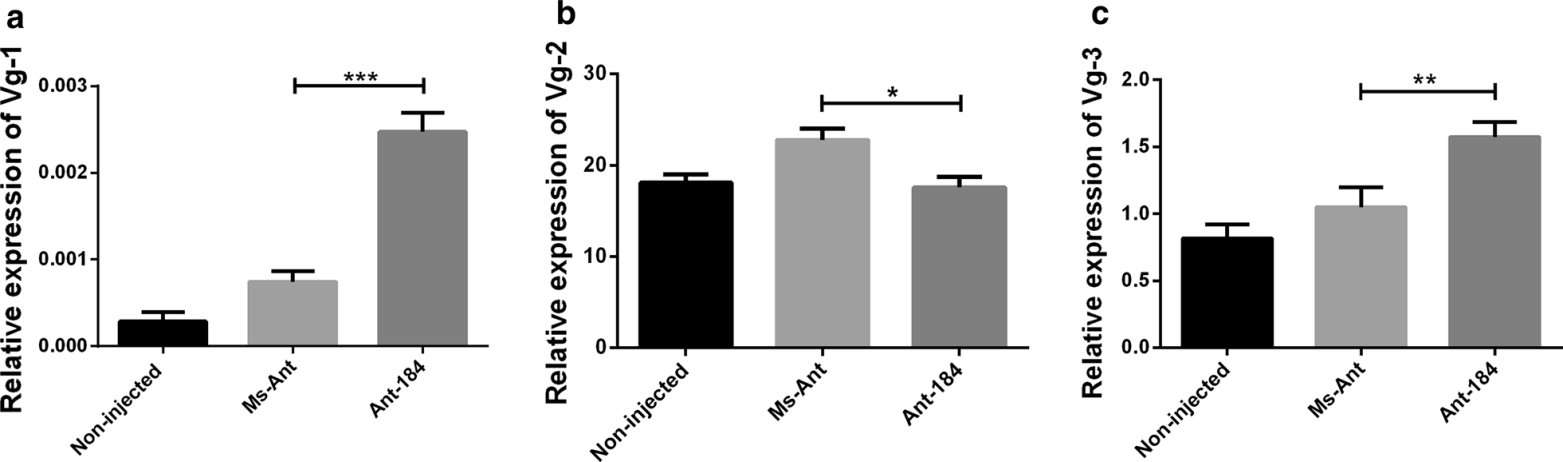
Relative expression of Vg proteins in semi-engorged ticks. Expression of Vg1 (**a**), Vg2 (**b**) and Vg3 (**c**) in treated (Ant-184) and control groups. Data represent three biological replicates with three technical replicates and are shown as the mean ± SEM

**Fig. 7:**
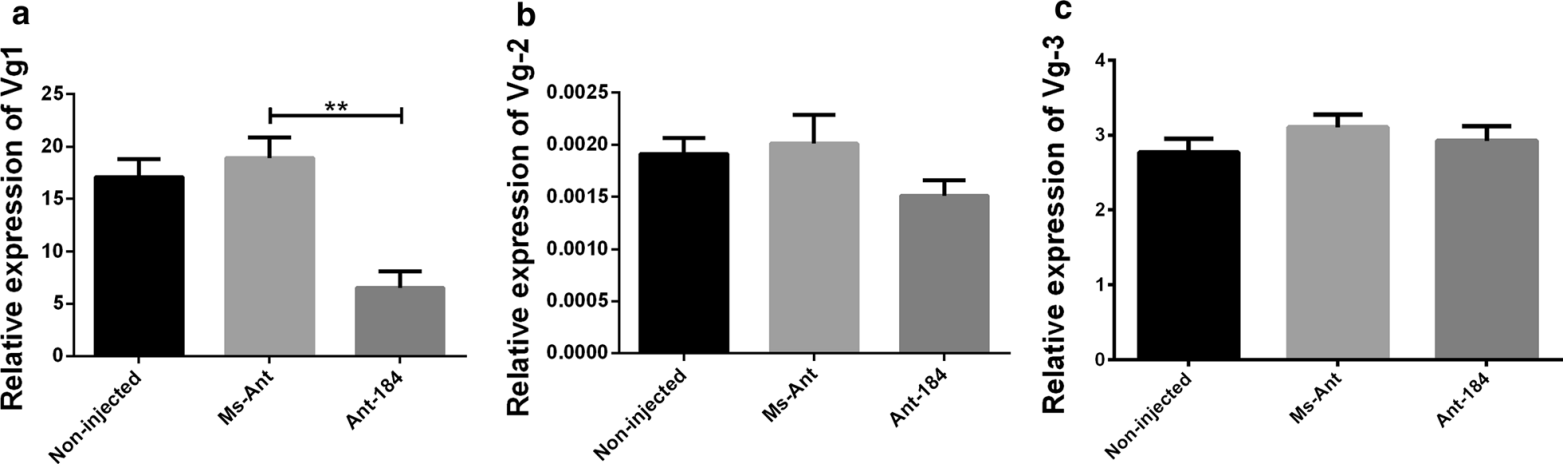
Relative expression of Vg proteins in engorged ticks. Expression of Vg1 (**a**), Vg2 (**b**) and Vg3 (**c**) in non-injected, Ms-Ant and Ant-184 groups. Data represent three biological replicates with three technical replicates and are shown as the mean ± SEM

**Fig. 8:**
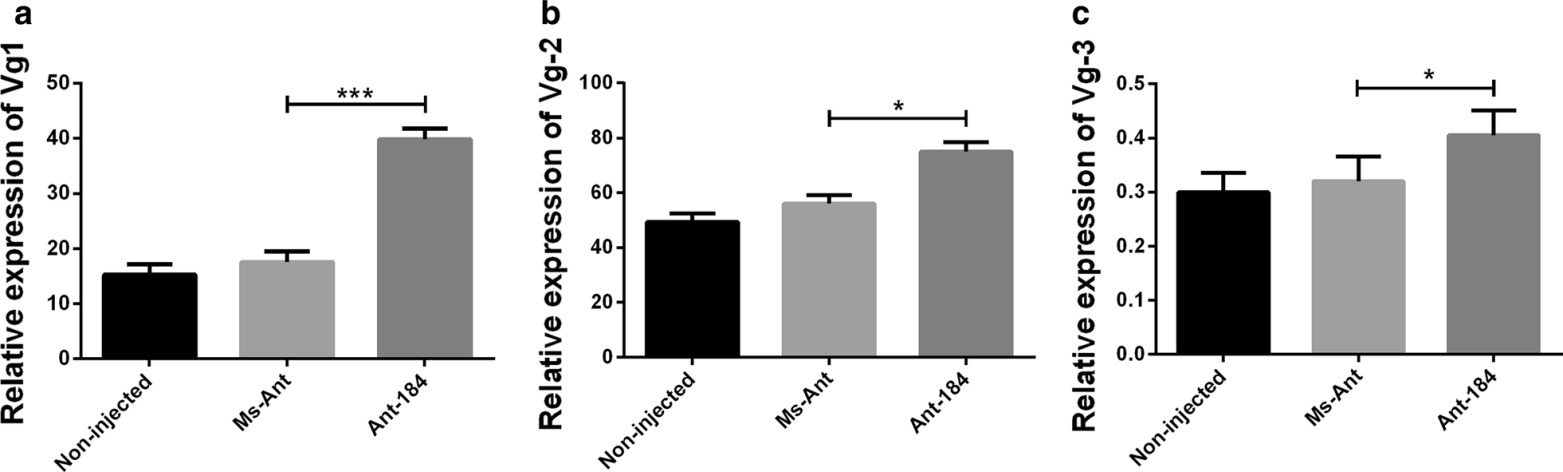
Relative expression of Vg proteins at day 2 after engorgement. Expression of Vg1 (**a**), Vg2 (**b**) and Vg3 (**c**) in non-injected, Ms-Ant and Ant-184 groups. Data represent three biological replicates with three technical replicates and are shown as the mean ± SEM

**Fig. 9:**
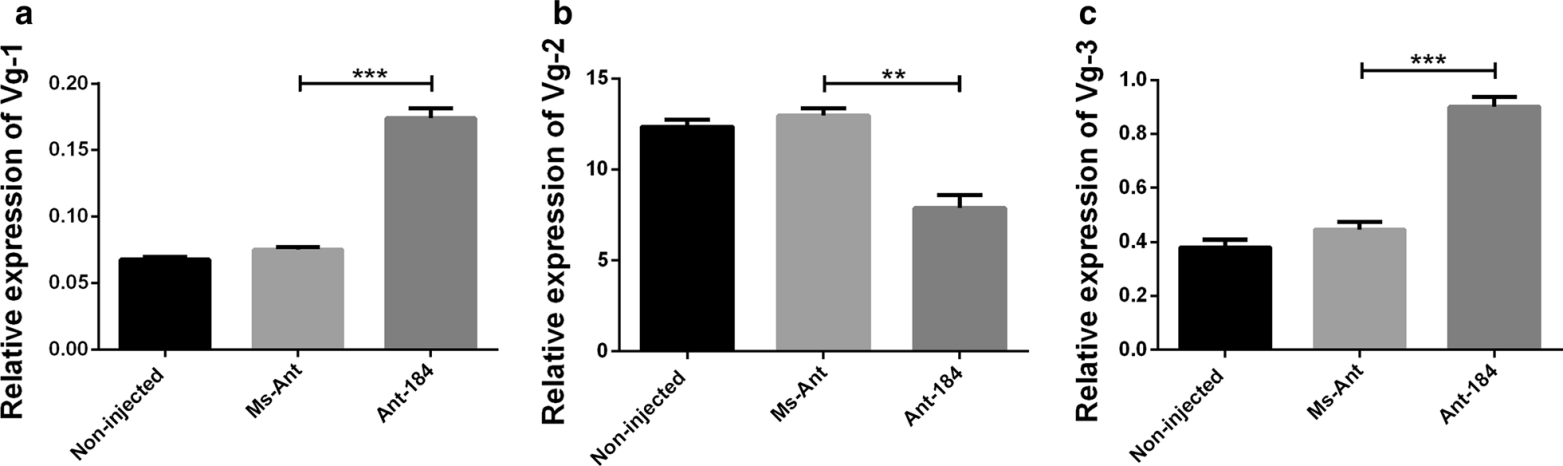
Relative expression of Vg proteins at egg stage after inhibition. Expression of Vg1 (**a**),Vg2 (**b**) and Vg3 (**c**) in non-injected, Ms-Ant and Ant-184 groups. Data represent three biological replicates with three technical replicates and are shown as the mean ± SEM

The relative expression of Vg1 (*t*_(4)_ = 29.51, *P* = 0.0001) and Vg3 (*t*_(4)_ = 5.259, *P* = 0.0061) was upregulated, while Vg2 (*t*_(4)_ = 3.867, *P* = 0.0180) was downregulated in semi-engorged ticks in Ant-184 group (Fig. 6). At engorgement, the relative expression of Vg1 (*t*_(4)_ = 5.133, *P* = 0.0068) was significantly lower compared to Vg2 and Vg3 in Ant-184, non-injected and Ms-Ant control (Fig. 7). At day 2 after engorgement, the relative expression levels of Vg1 (*t*_(4)_ = 10.64, *P* = 0.0004), Vg2 (*t*_(4)_ = 3.811, *P* = 0.0189) and Vg3 (*t*_(4)_ = 4.095, *P* = 0.0149) were upregulated in the treated group compared to control groups, in which Vg1 had the highest expression (Fig. 8). Vg expression was also measured in egg stages. Vg1 (*t*_(4)_ = 11.57, *P* = 0.0003) and Vg3 (*t*_(4)_ = 8.765, *P* = 0.0009) were upregulated, while Vg2 (*t*(4) = 6.213, *P* = 0.0034) was downregulated in Ant-184 compared to Ms-Ant and the non-injected control (Fig. 9).

## Discussion

miR-184 is evolutionarily conserved at the nucleotide level from insects to humans [22] with a single nucleotide difference (Table 5). In mosquitoes, several specific miRNA regulated blood digestion and egg development processes have been identified [23, 24]. There are a few studies on the miRNAs profiles in *R. sanguineus*, *R. haemaphysaloides*, *I. ricinus* and *H. longicornis* ticks [25–28]. Although various tick-specific miRNAs have been identified, functional studies have been reported only for miR-275 [16] and miR-375 [29] in *H. longicornis* and miR-79 in *I. scapularis* [30]. Since research on the expression profile of miR-184 in ticks had not been undertaken, we studied the expression of miR-184 at different growth phases of the tick life-cycle and in various tissues. The expression level was highest in unfed nymphs and lowest in unfed larvae (Fig. 1a). miR-184 was most highly expressed in midgut tissues. Expression was higher in fed than in unfed adult midgut and there was low expression in the salivary gland and ovary in both fed and unfed stages, respectively (Fig. 1b). Abundant expression of miR-184 in unfed nymphs and midgut of fed ticks suggests a role in development and maturation. Previous studies in *Drosophila* demonstrated that miR-184 has an important role in development [31] and has various functions in female germline [32].

**Table 5:** miR-184 mature sequences in different species

Species	miR-sequence
*Drosophila melanogaster*	Uggacggagaacugauaagggc
*Ixodes scapularis*	Uggacggagaacugauaagggc
*Homo sapiens*	Uggacggagaacugauaagggu
*Mus musculus*	Uggacggagaacugauaagggu

To explore the possible function of miR-184, Antagomir-184 was used to knock-down miR-184 in *H. longicornis* (Fig. 2). The engorged body weight was significantly reduced, while mean number of egg-laying days was significantly increased and the number of eggs, egg mass and hatchability were significantly reduced in treated group compared to the control groups. The expression of miR-184 was higher in unfed nymphs and the midgut of fed tick, and miR-184 silencing affected tick development. During oviposition, the eggs were also examined and the eggs in approximately 50% of Ant-184 group ticks became desiccated, lacked embryo development and failed to hatch (Fig. 5b, Table 3). Other research on miR-375 [16] and miR-275 in *H. longicornis* [29] showed that miRNA inhibition affected egg production, blood digestion, and oviposition.

Synthesis of the major egg yolk protein Vg and its uptake by oocytes are essential steps for egg maturation in all arthropods [12, 33]. Following vitellogenesis, Vg is released into the hemocoel filled with hemolymph and then is taken into developing oocytes *via* receptor-mediated endocytosis in ticks [34]. To study the silencing effect on oviposition, we predicted Vg (Vg1, Vg2, Vg3) proteins and analyzed their expression at different stages in Ant-184, non-injected and Ms-Ant groups. There was significant variation in the expression of Vg1, Vg2 and Vg3 in semi-engorged phase, ticks at day 2 after engorgement and egg stage in Ant-184, non-injected and Ms-Ant groups. Previous research on vitellogenin in *H. longicornis* showed that disruption in the profile of Vg proteins can affect egg development and oviposition [10].

This significant difference in the expression of Vg proteins at different phases after silencing of miR-184 suggests their involvement in duration of egg-laying, number of eggs laid, egg mass and hatchability.

## Conclusions

To our knowledge, this is the first information on the role of miR-184 in *H. longicornis*. Our data suggest that miR-184 targets Vg proteins and affects blood digestion and oviposition in *H. longicornis.*

## Data Availability

All data generated or analyzed during this study are included in the published article.

## Abbreviations

Vg: vitellogenin
PBS: phosphate-buffered saline
RH: relative humidity
Ant: antagomir
Ms-Ant: missense antagomir
